# Supplementary material for: Genome-scale phylogenetic analysis finds extensive gene transfer among fungi
Source: Philos Trans R Soc Lond B Biol Sci. 2015 Sep 26;370(1678):20140335. doi: 10.1098/rstb.2014.0335 (PMC4571573; doi:10.1098/rstb.2014.0335)
Supplement: Supplementary material for Genome-scale phylogenetic analysis finds extensive gene transfer among Fungi [file rstb20140335supp1.pdf]

Supplementary material for Genome-scale phylogenetic analysis finds extensive gene transfer among Fungi

Gergely J. Szöllősi[1], Adrián Arellano Davín[2], Eric Tannier[2,3], Vincent Daubin[2,4], Bastien Boussau[2,4]

July 14, 2015

[1]ELTE-MTA "Lendület" Biophysics Research Group, Pázmány P. stny. 1A., 1117 Budapest, Hungary;
[2]Laboratoire de Biométrie et Biologie Evolutive, Université de Lyon, F-69000 Lyon, France;
[3]Institut National de Recherche en Informatique et en Automatique Rhône-Alpes, F-38334 Montbonnot, France;
[4]Centre National de la Recherche Scientifique, Unité Mixte de Recherche 5558, Université Lyon 1, F-69622 Villeurbanne, France;

Corresponding author: Bastien Boussau, Laboratoire de Biométrie et Biologie Evolutive, Centre National de la Recherche Scientifique, Unité Mixte de Recherche 5558, Université Lyon 1, F-69622 Villeurbanne, France; Université de Lyon, F-69000 Lyon, France; E-mail: bastien.boussau@univ-lyon1.fr

Contents

1 Species trees **1**
1.1 Fungi 1
1.1.1 Tree A 1
1.1.2 Tree B 2
1.2 Cyanobacteria 4

2 Genome evolution in Ascomycota and Basidiomycota (tree B) **5**

1 Species trees

In this section we show the three species trees used in this work.

1.1 Fungi

1.1.1 Tree A

Tree A was obtained based on the Fungi concatenate.

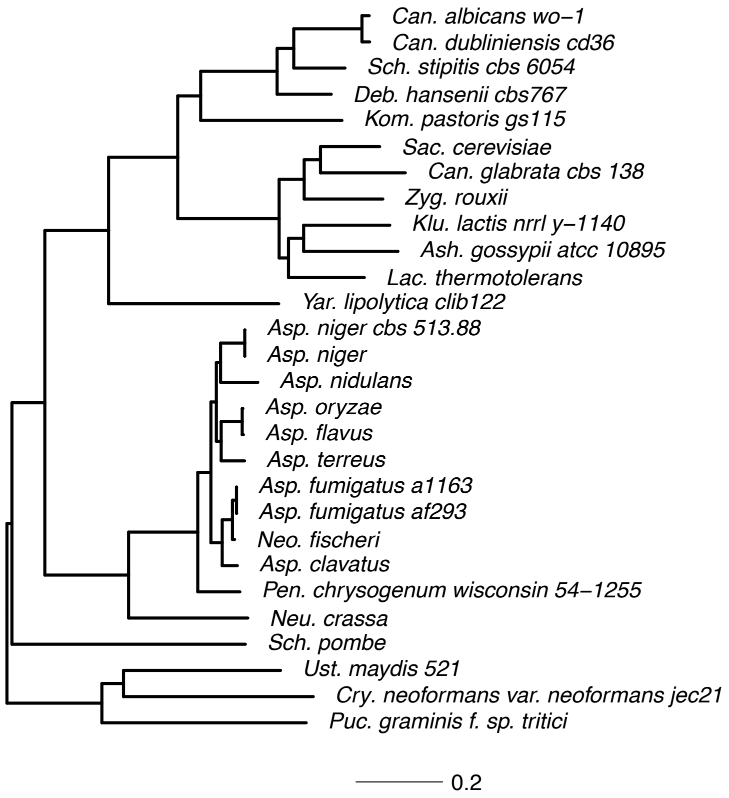

Figure 1: Tree A with branch lengths estimated by PhyML under model LG08 + Gamma.

1.1.2 Tree B

Tree B was obtained based on the literature.

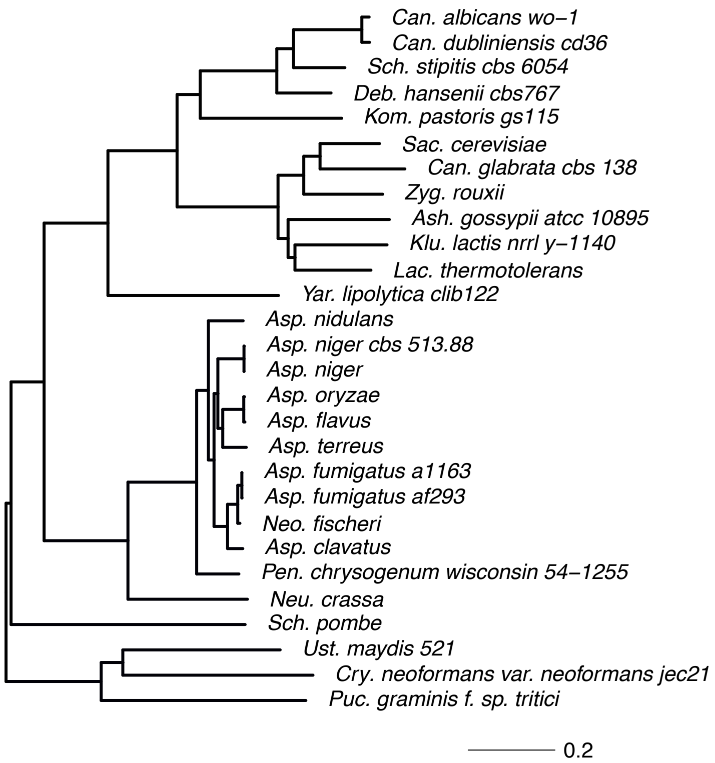

Figure 2: Tree B with branch lengths estimated by PhyML under model LG08 + Gamma.

1.2 Cyanobacteria

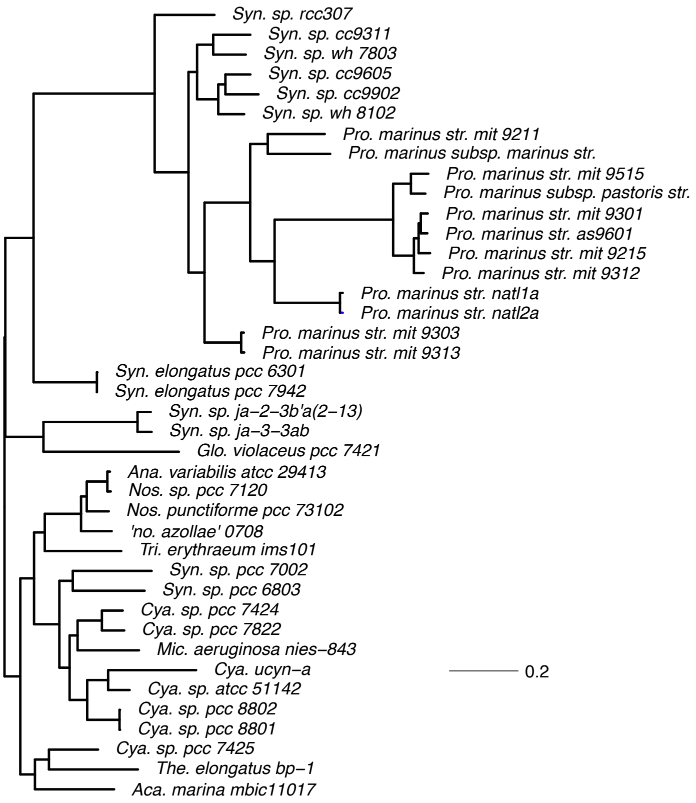

Figure 3: Tree of Cyanobacteria used in our analyses.

2 Genome evolution in Ascomycota and Basidiomycota (tree B)

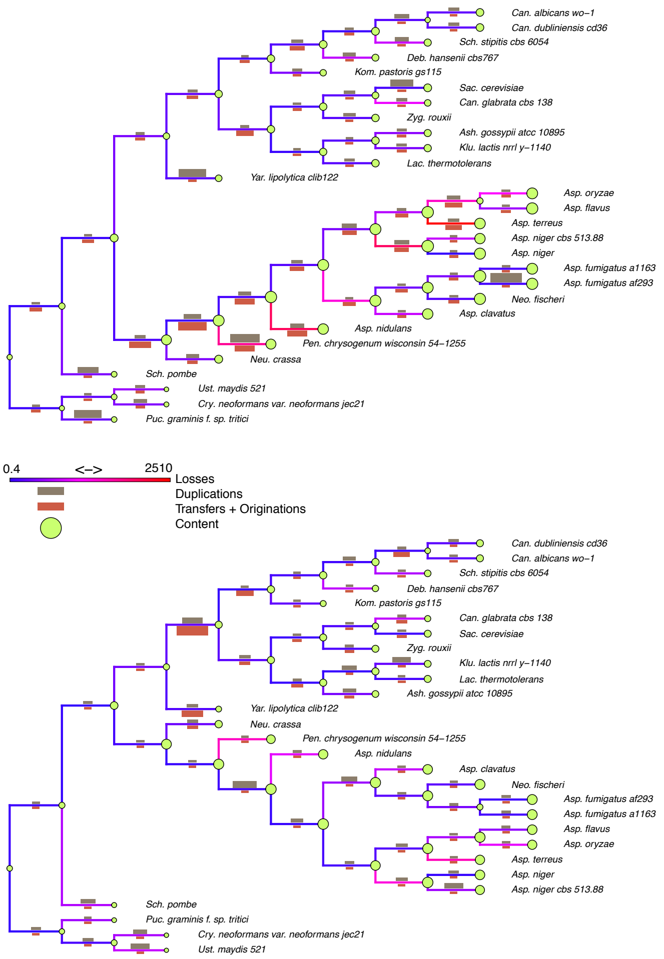

Figure 4: Genome evolution in Ascomycota and Basidiomycota (tree B). Edges are color-coded according to the inferred numbers of losses along the branches. Crimson bars represent numbers of gene gains (transfers + originations) arriving on the branch; taupe bars represent numbers of duplications happening on the branch. At each node, genome content size is represented as a green disk. Top: inferences from *ALEml_undated*. Bottom: inferences from *Count*.
